# Deletion of PGAM5 Downregulates FABP1 and Attenuates Long-Chain Fatty Acid Uptake in Hepatocellular Carcinoma

**DOI:** 10.3390/cancers15194796

**Published:** 2023-09-29

**Authors:** Ganesan Muthusamy, Chin-Chi Liu, Andrea N. Johnston

**Affiliations:** Louisiana State University School of Veterinary Medicine, Baton Rouge, LA 70803, USA; gmuthusamy@lsu.edu (G.M.); cliu@lsu.edu (C.-C.L.)

**Keywords:** liver cancer, long-chain fatty acids, fatty acid transporters

## Abstract

**Simple Summary:**

Hepatocellular carcinoma is the most common liver cancer. Metabolic dysfunction-associated steatotic liver disease is emerging as a primary cause of hepatocellular carcinoma. Lipid metabolism is altered in hepatocellular carcinoma to promote tumor growth. The mitochondrial membrane protein phosphoglycerate mutase 5 (PGAM5) is overexpressed in hepatocellular carcinoma and may play a role in lipid metabolism. The aim of our research study was to determine whether the expression of PGAM5 modified fatty acid uptake and lipid droplet accumulation in hepatocellular carcinoma cells. We learned that by eliminating PGAM5 expression, lipid droplet accumulation was reduced in hepatocellular carcinoma and that this may be due to reduced fatty acid uptake. Enhanced understanding of fatty acid regulation in cancer supports the rationale design of therapeutics targeting metabolic pathways.

**Abstract:**

Phosphoglycerate mutase 5 (PGAM5) is a Ser/His/Thr phosphatase responsible for regulating mitochondrial homeostasis. Overexpression of PGAM5 is correlated with a poor prognosis in hepatocellular carcinoma, colon cancer, and melanoma. In hepatocellular carcinoma, silencing of PGAM5 reduces growth, which has been attributed to decreased mitophagy and enhanced apoptosis. Yet in colon cancer, PGAM5’s pro-tumor survival effect is correlated to lipid metabolism. We sought to identify whether deletion of PGAM5 modulated lipid droplet accrual in hepatocellular carcinoma. HepG2 and Huh7 *PGAM5* knockout cell lines generated using CRISPR/Cas9 technology were used to measure cell growth, cellular ATP, and long-chain fatty acid uptake. Expression of hepatocellular fatty acid transporters, cluster of differentiation 36 (CD36), solute carrier family 27 member 2 (SLC27A2), solute carrier family 27 member 5 (SLC27A5), and fatty acid binding protein 1 (FABP1) was measured by quantitative PCR and Western blot. We found that deletion of PGAM5 attenuates hepatocellular carcinoma cell growth and ATP production. Further, *PGAM5* knockout ameliorates palmitate-induced steatosis and reduces expression of FABP1 in HepG2 and Huh7 cell lines. PGAM5’s role in hepatocellular carcinoma includes regulation of fatty acid metabolism, which may be related to expression of the fatty acid transporter, FABP1.

## 1. Introduction

Hepatocellular carcinoma (HCC) is the fourth leading cause of cancer death worldwide [1]. Epidemiological estimates indicate that greater than 1 million people will be diagnosed with HCC by 2025 [1,2]. Overexpression of tumoral phosphoglycerate mutase 5 (PGAM5) is correlated with reduced survival in hepatocellular carcinoma, as well as other cancers [3,4,5,6,7,8]. The mitochondrial membrane protein PGAM5 is a serine/threonine/histidine phosphatase with a diverse set of substrates [6,7,8,9,10,11]. Among these are mediators of mitophagy and multiple regulated cell death pathways [12,13,14,15,16,17]. PGAM5 can inhibit and potentiate apoptosis dependent on the phosphorylation state of the anti- apoptotic protein, B-cell lymphoma-extra large (Bcl_X_L) [18]. Aside from its phosphatase activity, PGAM5 also promotes the stabilization of proteins at the mitochondrial membrane. PGAM5 plays a role in oxeiptosis through its interaction with the oxidant sensor KEAP1 [19]. PGAM5 tethers KEAP1 to the mitochondrial membrane. When moderate oxidative damage occurs, the KEAP1–NRF2 complex dissociates, allowing translocation of the antioxidant transcription factor NRF2 to the nucleus. In cases of extreme oxidant injury, PGAM5 dissociates from KEAP1 and interacts with and dephosphorylates the mediator of caspase independent cell death apoptosis inducing factor-1 (AIFM1), resulting in caspase-independent, immunologically silent cell death. PGAM5 also enhances mitochondrial fission of damaged mitochondria through dynamin-related protein 1 (Drp1) dephosphorylation [20]. PGAM5’s inhibitory role in apoptosis through Bcl_X_L dephosphorylation and enhancement of Drp1-mediated mitophagy have been correlated to its pro-cancer survival effect, wherein *PGAM5* knockdown has been shown to attenuate hepatocellular tumor growth in vitro and in xenograft models of HCC [6,7,21].

In colon cancer, PGAM5 has been linked to tumor lipid metabolism, which has not been explored in HCC [5]. Metabolic reprogramming is a hallmark of oncogenesis [22,23]. Dysregulated growth imparts unique nutrient requirements. When glucose and protein stores are depleted, lipids provide an energetically dense fuel source. Further, lipid droplets serve as depots for membrane repair components and metabolites needed for cell signaling. In cancer, the intracellular lipid pool is replenished by upregulation of de novo lipogenesis and altered fatty acid uptake [23]. Long-chain fatty acid (LCFA) uptake in hepatocytes is enabled by cluster of differentiation 36 (CD36) fatty acid translocase, the fatty acid transport proteins of the solute transporter family (solute carrier family 27 member 2 (SLC27A2), solute carrier family 27 member 5 (SLC27A5)), and the fatty acid binding proteins, predominantly fatty acid binding protein 1 (FABP1). Fatty acid transporters are overexpressed in many cancers and are known to promote tumor growth [24,25,26].

The purpose of this study was to determine if loss of PGAM5 reduced HCC viability and disrupted fatty acid uptake. We found that deletion of PGAM5 mitigates hepatocellular growth and ATP production in HCC cells. Treatment with the long-chain fatty acid palmitate upregulates *PGAM5* mRNA expression and knockout of *PGAM5* attenuates palmitate-induced steatosis. Finally, we identified downregulated expression of FABP1 in HCC cells. *PGAM5* knockout alters the lipid metabolic phenotype in HCC cells.

## 2. Materials and Methods

### 2.1. Cell Lines and Culture Conditions

The HepG2 cell line (HB-8065) was purchased from ATCC^®^. The Huh-7D 12 (Huh7, 01042712) cell line was purchased from Millipore Sigma. The HepG2 *PGAM5* knockout cell line was generated and validated by Synthego Corporation (Redwood City, CA, USA). The Huh-7D *PGAM5* knockout line was generated in our laboratory using *PGAM5* CRISPR/Cas9 KO Plasmid (sc-401300, Santa Cruz Biotechnology, Inc., Dallas, TX, USA). Following transfection, cell cultures were diluted to enable single cell per well plating. Single cells expressing green fluorescent protein indicative of plasmid transfection were clonally expanded and screened for loss of PGAM5 protein expression by Western blot. A single clone with loss of PGAM5 protein expression was utilized for all experiments. The HepG2 and Huh7 cell lines were maintained in DMEM, high glucose media (25 mmol/L glucose, Gibco, Grand Island, NY, USA) supplemented with 10% fetal bovine serum (Avantor, Radnor, PA, USA), and 1% Penicillin–Streptomycin (Gibco). Nutrient depleted media were composed of DMEM (glucose 12.5 mol/L), FBS 1%, and L-Carnitine 0.5 mM. Bovine serum albumin 80 µM (BSA; 28556, Cayman Chemical, Ann Arbor, MI, USA) and BSA–Palmitate Saturated Fatty Acid Complex 500 µM (29558, Cayman Chemical) were applied as indicated.

### 2.2. Cell Growth and ATP Quantification Assays

Live cell imaging was used for label-free, cell growth quantification of bright field area Incucyte^®^ S3 Live Cell Analysis Instrument (Sartorius, Goettingen, Germany). ATP was quantified using the CellTiter-Glo assay (Promega, Madison, WI, USA), which was performed according to the manufacturer’s instructions. Luminescence was recorded with a Synergy LX multimode reader (Bio-Tek, Winooski, VT, USA). Three experiments with eight replicates per experiment were completed for cell growth and ATP quantification assays.

### 2.3. Quantification of Fatty Acid Uptake

Cell culture wells were washed twice with PBS and fixed in 10% formalin for 30 min at room temperature. Formalin was removed and cells were washed twice with deionized water and treated with 60% isopropanol for 5 min. Oil Red O solution was applied to each well for 20 min rotating at room temperature and wells were subsequently washed 3 times with deionized water. Total cell area was evaluated using the Incucyte^®^ S3 Live Cell Analysis Instrument (Sartorius) to confirm equal cell density prior to fatty acid extraction. Plates were washed 3 times with 60% isopropanol for 5 min rotating at room temperature. Oil Red O was extracted with 100% isopropanol for 5 min and absorbance was measured at 492 nm on the Synergy LX multimode reader (Bio-Tek, Winooski, VT, USA). Quantification of fatty acids was performed in 3 experiments with a minimum of 2 replicates.

### 2.4. Quantitative PCR

Total RNA from cultured cells was extracted using the RNeasy kit (Qiagen, Germantown, MD, USA) according to manufacturer’s instructions and quantified using a Nanodrop™ (Thermo Fisher, Waltham, MA, USA). Reverse transcription and qPCR were performed per manufacturer’s protocols (qScript™ cDNA SuperMix and PerfeCTaSYBR^®^ Green FastMix^®^ Reaction Mix, QuantaBio, Beverly, MA, USA). The housekeeping genes 18s and ß-Actin were used for standardization of sample mRNA content. Analysis was performed on 7900 HT Applied BioSystems™ Real-Time PCR Detection System (BioRad Bedford, MA, USA). Each quantitative RT-PCR was performed in duplicate with 12.5 ng cDNA. The relative expression levels of the target genes were calculated and expressed as -ddCt relative to 18S RNA expression in wild type cells. All primer sequences are listed in Supplementary Table S1. Quantitative PCR was run in duplicate in each of the 3 experiments.

### 2.5. Western Blotting

Cell pellets were lysed with five volumes of lysis buffer (50 mM Tris (pH 7.4), 137 mM NaCl, 1 mM EDTA, 1% Triton X-100, and 10% glycerol, supplemented with 1× halt protease inhibitor cocktail (ThermoFisher, Waltham, MA, USA)) and incubated on ice for 30 min. Cell lysates were centrifuged at 20,000× *g* for 15 min, and the supernatant was collected. The protein content was measured and normalized using a BCA Protein Assay Kit (ThermoFisher, Waltham, MA, USA). Total proteins (20–40 μg) were separated by SDS-PAGE and transferred onto PVDF membranes. After blocking with 5% non-fat milk, the membranes were incubated with primary antibodies at 4 °C overnight and subsequently incubated with their corresponding HRP-labeled secondary antibodies. Bands were detected using ECL (RPN2106, Cytvia, Marlborough, MA, USA) on myECL Imager (G2236X, Thermo Fisher, Waltham, MA, USA). The protein relative intensity was analyzed using ImageJ software (National Institutes of Health, Bethesda, MD, USA). Primary antibodies: Actin (Invitrogen, Waltham, MA, USA; AB_2223496), CD36 (Invitrogen, Waltham, MA, USA; AB_2807046), SLC27A2 (Invitrogen, AB_2851750), SLC27A5 (Invitrogen, AB_2786966), FABP1 (LSBio, Shirley, MA, USA; LS-C369600), PGAM5 (Invitrogen, Waltham, MA, USA; AB_2900380). Secondary antibodies: Goat anti-Rabbit HRP (Invitrogen, Waltham, MA, USA; AB_228341), Anti-mouse (Invitrogen, Waltham, MA, USA; AB_228295). Western blot was performed on cell lysates from 3 non-replicated experiments per cell line.

### 2.6. Statistical Analyses

Statistical analyses were performed using JMP Pro 16.2.0 (SAS Institute, Cary, NC, USA) and figures were made using GraphPad Prism, version 9.4 (GraphPad Prism Software, Inc., La Jolla, CA, USA). Data are expressed as mean ± standard deviation (SD), standard error of the mean (SEM), or confidence interval (CI) as indicated. Logarithmic transformation was performed for data that did not meet the normality criteria. Normality of residuals from the models were accessed and confirmed via Shapiro–Wilk tests. A mixed analysis of variance (ANOVA) model was used to analyze -ddCT data (qPCR) with wild type and PGAM5 knockout as the fixed effect and each plate as the random effect. Cellular growth, ATP production, and fatty acid uptake were measured by a 2-way mixed ANOVA with cell type, time, and their interaction as the fixed effect; where appropriate, a post hoc Tukey test was used for comparisons between groups. Paired *t*-tests were used for comparisons of protein expression. Differences were considered significant at *p* < 0.05.

## 3. Results

### 3.1. Deletion of PGAM5 Attenuates Hepatocellular Carcinoma Cell Growth

**Figure 1:**
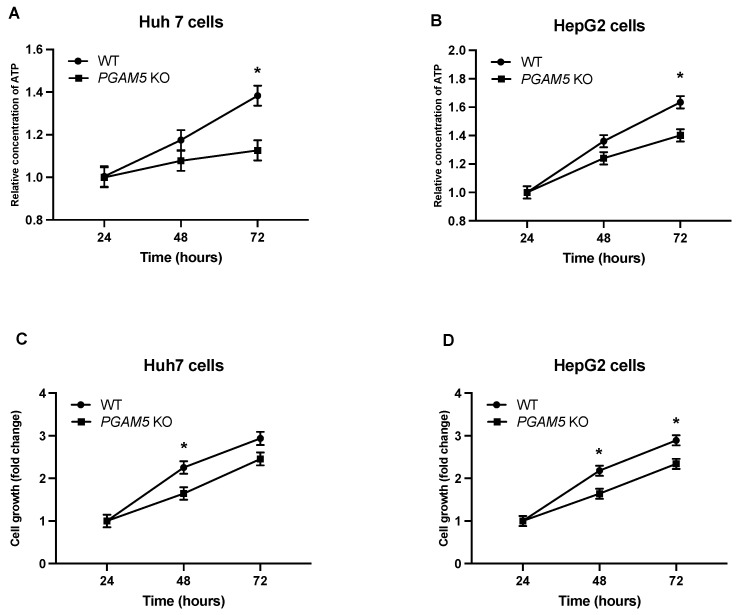
ATP production and cell growth are attenuated with ablation of *PGAM5*. (**A**) Huh7 wild type (WT) and *PGAM5* knockout cells (KO) cells (1 × 10^5^) were cultured under standard conditions. ATP concentration was measured at 24 h, 48 h, and 72 h. Values were normalized to the 24 h measurement. The mean values and SEMs (*n* = 3 experiments, 8 replicates per experiment) are indicated. (**B**) HepG2 WT and *PGAM5* KO cells (1 × 10^5^) were cultured under standard conditions. ATP concentration was measured at 24 h, 48 h, and 72 h. Values were normalized to the 24 h measurement. The mean values and SEMs (*n* = 3 experiments, 8 replicates per experiment) are displayed. (**C**) Huh7 WT and *PGAM5* KO cells (1 × 10^5^) were cultured under standard conditions. Bright field cell area was quantified at 24 h, 48 h, and 72 h. Values were normalized to the 24 h measurement. The mean values and SEMs (*n* = 3 experiments, 8 replicates per experiment) are indicated. (**D**) HepG2 WT and *PGAM5* KO cells (1 × 10^5^) were cultured under standard conditions. Bright field cell area was quantified at 24 h, 48 h, and 72 h. Values were normalized to the 24 h measurement. The mean values and SEMs (*n* = 3 experiments, 8 replicates per experiment) are indicated, * *p* < 0.05.

To evaluate the effects of *PGAM5* deletion on HCC cell growth and viability, Huh7 and HepG2 were cultured under standard conditions for 72 h. ATP production was measured concurrently (Figure 1A,B). Although the *PGAM5* knockout cells remained viable, ATP production was significantly reduced. Consistent with previous reports, cell growth was attenuated in Huh7 and HepG2 PGAM5 knockdown cell lines (Figure 1C,D) [7].

### 3.2. Long-Chain Fatty Acid-Induced Steatosis Is Ameliorated with PGAM5 Knockout

**Figure 2:**
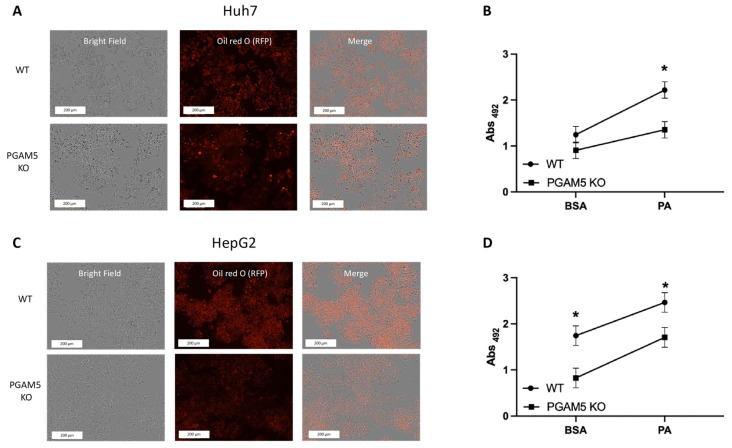
Effect of PGAM5 deletion on long-chain fatty acid uptake. (**A**) Huh7 wild type (WT) and *PGAM5* knockout cells (KO) cells (5 × 10^5^) were cultured under standard conditions for 24 h. Cells were treated with bovine serum albumin (BSA) or palmitate (PA) for 16 h under nutrient-depleted conditions. Representative images of ORO-stained (red) cells validating equal cell density. Bars in the images represent 200 μm. (**B**) Fatty acid uptake was quantified spectrophotometrically by Oil Red O (ORO) Absorbance (Abs) at 492 nm in Huh7 cells. The mean values and SEMs (*n* = 3 experiments with a minimum of 2 replicates) are indicated. (**C**) HepG2 WT and *PGAM5* KO cells (5 × 10^5^) were cultured under standard conditions for 24 h. Cells were treated with BSA or PA for 16 h under nutrient depleted conditions. Representative images of ORO-stained (red) cells validating equal cell density. (**D**) Fatty acid uptake was quantified spectrophotometrically following ORO staining in HepG2 cells. The mean values and SEMs (*n* = 3 experiments with a minimum of 2 replicates) are indicated, * *p* < 0.05.

To determine if *PGAM5* deletion impacts lipid droplet accumulation, WT and *PGAM5* knockout HCC cell lines were treated overnight with palmitate. Lipid droplet accrual was assessed by Oil Red O spectrophotometric quantitation and cell density was assessed by image analysis (Figure 2). Palmitate-induced steatosis was significantly reduced in *PGAM5* KO compared to WT cell lines (Figure 2B,D).

### 3.3. Long-Chain Fatty Acid Treatment Upregulates PGAM5 mRNA Expression

**Figure 3:**
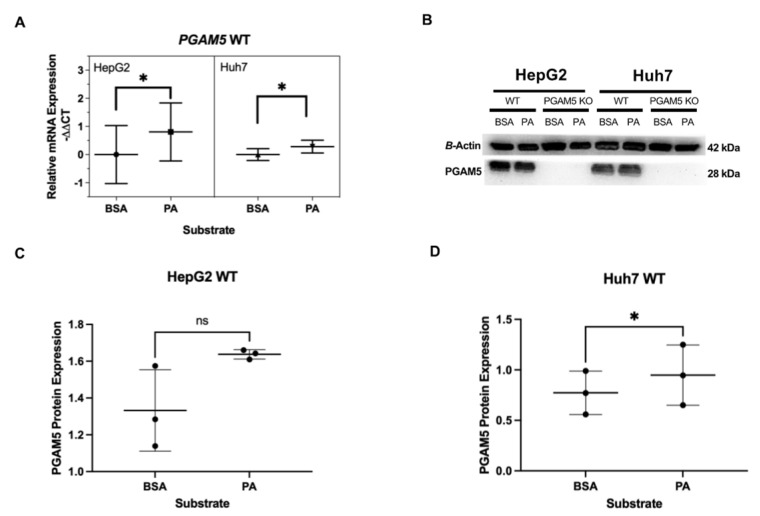
Effect of long-chain fatty acid treatment on *PGAM5* expression. (**A**) Messenger RNA expression in HepG2 wild type (WT) and Huh7 WT cells treated with bovine serum albumin (BSA) or palmitate (PA) was determined by real-time qPCR. The mean normalized −ΔΔCt values and CI (*n* = 3 experiments with 2 replicates per experiment) are displayed for *PGAM5.* (**B**) Protein expression in HepG2 WT, HepG2 *PGAM5* KO, Huh7 WT, and Huh7 *PGAM5* KO cells treated with BSA or PA was determined by Western blot (*n* = 3 experiments). Representative immunoblots of PGAM5 and ß-actin are displayed. Protein expression was quantified by densitometry for (**C**) HepG2 and (**D**) Huh7 cells. The mean protein expression and SDs are displayed (bars) with individual experimental values as dots, * *p* < 0.05.

To determine if long-chain fatty acids influence PGAM5 expression, HepG2 and Huh7 cells cultured under standard conditions were treated overnight with palmitate or bovine serum albumin (BSA) as a control. Cells were harvested and mRNA and protein were extracted. Messenger RNA and protein expression were quantified with qPCR and Western blot, respectively. PGAM5 mRNA expression was upregulated in WT HepG2 and WT Huh7 cell lines following palmitate treatment (Figure 3A). A minimal but statistically significant upregulation in PGAM5 protein expression was identified in Huh7 cells but the difference in expression was not statitically significant in HepG2 cells following palmitate treatment (Figure 3B–D, Supplementary Figures S1–S3).

### 3.4. Hepatocellular Fatty Acid Transporter Expression Is Downregulated with PGAM5 Knockout

**Figure 4:**
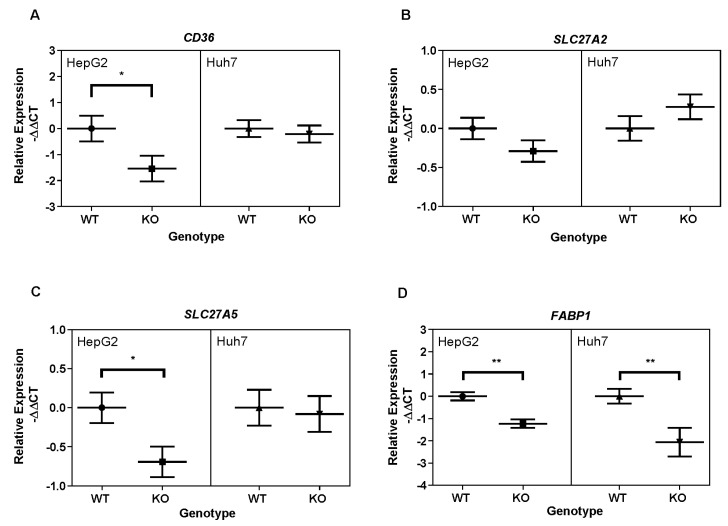
Effect of PGAM5 deletion on hepatocellular fatty acid transporter gene expression. Messenger RNA expression in HepG2 wild type (WT, dots), HepG2 *PGAM5* knockout (KO, squares), Huh7 WT (triangle), and Huh7 *PGAM5* KO (inverted triangle) cells was determined by real-time PCR. The mean normalized −ΔΔCt values and CIs (*n* = 3 experiments with 2 replicates per experiment) are displayed for (**A**) *CD36*, (**B**) *SLC27A2*, (**C**) *SLC27A5*, and (**D**) *FABP1*. * *p* < 0.05, ** *p* < 0.01.

Next, we investigated the effects of *PGAM5* knockout on mRNA expression of the liver fatty acid transporter proteins FABP1 in the fatty acid transport family, and SLC27A2, SLC27A5, and CD36 in the solute carrier family (Figure 4A–D). Messenger RNA and protein expression were quantified with qPCR and Western blot, respectively. In HepG2 *PGAM5* knockout cells, gene expression of *CD36, SLC27A5*, and *FABP1* was significantly downregulated compared to wildtype cells, whereas in Huh7 cells, only *FABP1* gene expression was significantly downregulated (Figure 4A,C,D; Supplementary Tables S2 and S3).

CD36, SLC27A5, and SLC27A2 protein expression were not significantly different between groups (Figure 5A–D; Supplementary Figures S4–S10). FABP1 protein expression was significantly reduced in both HepG2 and Huh7 PGAM5 knockout cells (Figure 5A,F; Supplementary Figures S4–S6).

## 4. Discussion

Manipulation of lipid metabolism is a cancer survival mechanism. Enhanced systemic fatty acid accumulation imparts a protective effect, insulating the cancer cell from oxidant damage to lipid bilayers and providing an alternate source of ATP under nutrient-depleted conditions [22,23,25,26]. In this study, we revealed that *PGAM5* knockout ameliorates lipid droplet accumulation in the HepG2 and Huh7 cell lines. PGAM5 is a member of the phosphoglycerate mutase (PGAM) superfamily. PGAM family members share a conserved PGAM domain, which catalyzes transfer of phospho groups on phosphoglycerates [27]. Phosphoglycerate mutase 1, 2, and 4 (PGAM1, PGAM2, PGAM4) catalyze conversion of 3-phosphoglycerate to 2-phosphoglcerate in the glycolytic pathway [28]. Additional PGAM family members, the 6-phosphofructo-2-kinase/fructose-2,6-biphosphatases (PFKFB), also play roles in the regulation of glucose metabolism [29]. Although PGAM5 shares the RHGE catalytic motif, PGAM5 does not have phosphotransferase or hydrolase activity [30].

We have identified that the fatty acid transporter FABP1 is downregulated secondary to *PGAM5* knockout. This is the first time that PGAM5 expression has been correlated to exogenous fatty acid import in hepatocellular carcinoma. FABP1, also known as Liver-FABP (L-FABP), mediates uptake, intracellular mobilization, and metabolism of long-chain fatty acids [31,32]. FABP1 deletion inhibits fatty acid uptake and alters its distribution in cultured primary hepatocytes and murine models [33,34,35,36]. FABP1 is located predominantly in the cytosol but to a lesser extent is present in the nucleus and on the mitochondrial outer membrane [37]. Aside from long-chain fatty acids, FABP1 binds a host of other ligands including acyl-CoAs, bile acids, bilirubin, endocannabinoids, and heme [32,38,39]. As a mediator of intracellular trafficking of free fatty acids, FABP1 limits the detergent effects of these moieties [40]. Fatty acid binding proteins can also directly scavenge free radicals and limit lipotoxicity [41,42]. PGAM5′s influence of fatty acid transport complements its regulation of mitophagy, which is critical to maintaining mitochondrial homeostasis in the oxidative intracellular environment [4,7,12,21].

Overexpression of FABP1 is identified in colorectal adenocarcinomas, cholangiocarcinomas, and HCC [43]. In HCC, FABP1 promotes angiogenesis and migration mediated by vascular endothelial growth factor A (VEGF-A) [44]. VEGF-A is induced through the Akt/mTOR/P70S6K pathway in a hypoxia inducible factor 1 α (HIF-1α)-dependent manner. Further, in WT HepG2 and Huh7 cells, *PGAM5* mRNA expression is upregulated following treatment with the long-chain fatty acid, palmitate; however, only Huh7 cells demonstrate a significant increase in PGAM5 protein levels. Additional experiments are needed to determine if PGAM5 expression level or phosphatase activity is relevant to PGAM5’s role in tumoral fatty acid metabolism specifically in metabolic disease associated HCC. Based on the results presented herein, the mechanism by which PGAM5 influences FABP1 expression may involve transcriptional regulation. Transcriptional regulation of FABP1 is complex, involving CCAAT enhancer binding protein α (C/EBPα) repression and forkhead box protein A1 (FOXA1)/peroxisome proliferator-activated α (PPARα) activation [45]. It is tempting to consider whether genetic manipulation of *PGAM5* could be used therapeutically in HCC [6,46]. Yet, many questions need to be answered before undertaking this endeavor in the clinical setting [47]. First, it remains to be determined how loss of PGAM5 interferes with FABP1 expression and how the PGAM5 and FABP1 pathways converge to impart a pro-cancer survival benefit in HCC. This work did not specifically assess whether PGAM5 knockout represses transcription of *FABP1*. Future experiments using chemical inhibition of transcription are required to test this hypothesis. Additionally, animal modeling is required to determine if the metabolic changes associated with *PGAM5* knockout observed in cell culture persist in vivo.

## 5. Conclusions

Our data suggest that PGAM5’s role in hepatocellular carcinoma includes regulation of fatty acid metabolism, which may be implemented through expression of the fatty acid transporter, FABP1.

## Figures and Tables

**Figure 5:**
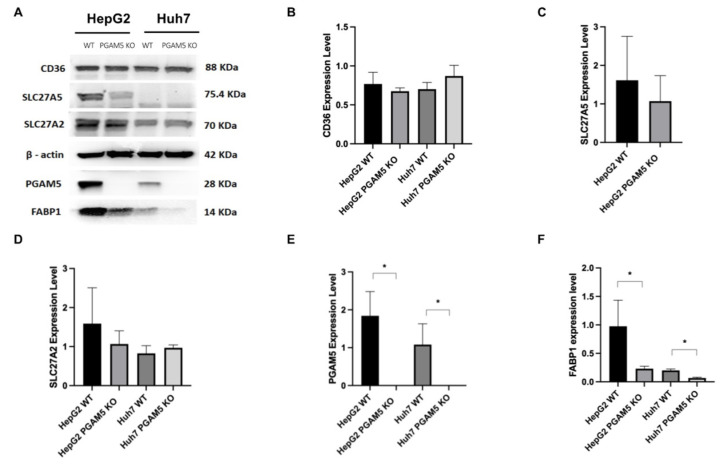
Effect of PGAM5 deletion on hepatocellular fatty acid transporter protein expression. (**A**) Fatty acid transporter protein expression in HepG2 WT, HepG2 *PGAM5* KO, Huh7 WT, and Huh7 *PGAM5* KO cells was determined by Western blot (*n* = 3 experiments). Representative immunoblots of CD36, SLC27A5, SLC27A2, ß-actin, PGAM5, and FABP1 are displayed. (**B**) Protein expression was quantified by densitometry (ImageJ). Mean relative values ± SDs normalized to ß-actin are indicated for CD36, (**C**) SLC27A5, (**D**) SLC27A2, (**E**) PGAM5, and (**F**) FABP1, * *p* < 0.05.

## Data Availability

All data generated or analyzed during this study are included within the article.

## Supplementary Materials

The following supporting information can be downloaded at: https://www.mdpi.com/article/10.3390/cancers15194796/s1, Figure S1: PGAM5 and actin expression in HepG2 and Huh7 cells. Figure S2: PGAM5 and actin expression in HepG2 and Huh7 cells. Figure S3: PGAM5 and actin expression in HepG2 and Huh7 cells. Figure S4: CD36, PGAM5, actin, and FABP1 expression in HepG2 and Huh7 cells; Figure S5: CD36, SLC27A2, PGAM5, actin, and FABP1 expression in HepG2 and Huh7 cells; Figure S6: Actin, SLC27A2, and FABP1 expression in HepG2 and Huh7 cells; Figure S7: SLC27A5 and actin expression in HepG2 and Huh7 cells; Figure S8: SLC27A5 and actin expression in HepG2 and Huh7 cells; Figure S9: SLC27A5 and actin expression in HepG2 and Huh7 cells; Figure S10: CD36 and actin expression in HepG2 and Huh7 cells; Table S1: Quantitative PCR primers; Table S2: Individual qPCR data points corresponding to Figure 3; Table S3: Individual qPCR data points corresponding to Figure 3.
