# Supplementary material for: Deletion of PGAM5 Downregulates FABP1 and Attenuates Long-Chain Fatty Acid Uptake in Hepatocellular Carcinoma
Source: Cancers (Basel). 2023 Sep 29;15(19):4796. doi: 10.3390/cancers15194796 (PMC10571733; doi:10.3390/cancers15194796)
Supplement: Supplementary file 1 [file cancers-15-04796-s001.zip › cancers-2559732-supplementary.pdf]

Supplementary Figure S1. PGAM5 and Actin Expression in HepG2 and Huh7 cells. Protein expression of Actin (42 kDa) and PGAM5 (28 kDa) are displayed on the immunoblot (left). The bright field image of the top PVDF membrane is displayed on the right. **B.** The bright field image of the individual and assembled PVDF membranes are displayed on the right. BSA: bovine serum albumin, PA: palmitate. WT: wild type, KO: *PGAM5* KO

A

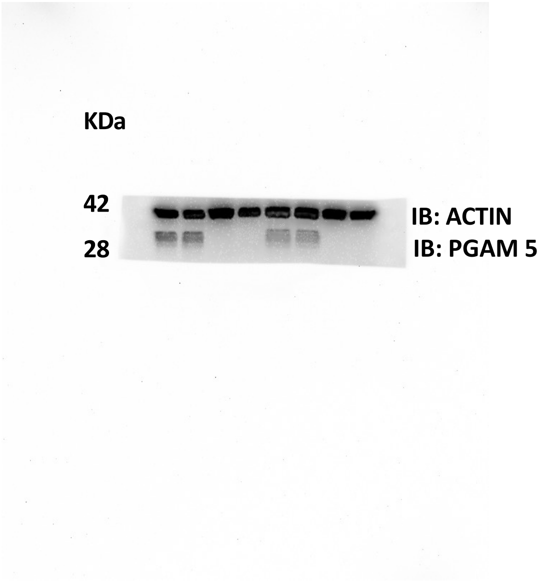

B

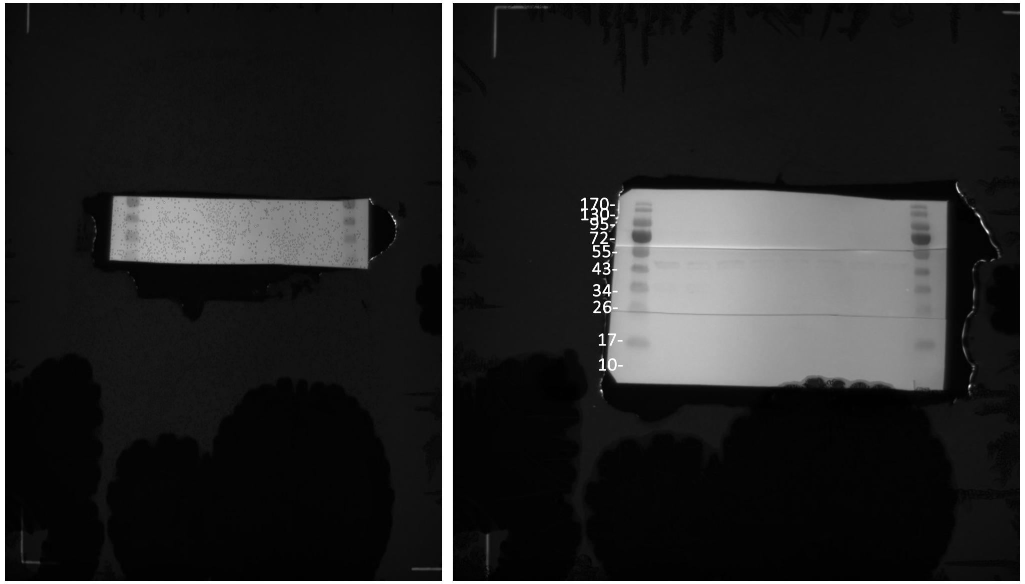

Lane 1 – Marker

Lane 2 – HepG2 WT – BSA

Lane 3 - HepG2 WT – PA

Lane 4 - HepG2 – PGAM5 KO - BSA

Lane 5 - HepG2 – PGAM5 KO - PA

Lane 6 – Huh7 WT – BSA

Lane 7 - Huh7 WT - PA

Lane 8 – Huh7 – PGAM5 KO – BSA

Lane 9 – Huh7 – PGAM5 KO – PA

Lane 10 - Marker

Densitometry Readings

	HepG2 WT		HepG2 KO		Huh7 WT		Huh7 KO	
	BSA	PA	BSA	PA	BSA	PA	BSA	PA
ACTIN	7314.497	5623.891	8148.497	5918.426	7056.397	5776.184	5462.962	6774.61
PGAM5	8333.184	9051.426	0	0	5434.326	5459.397	0	0

Relative Intensities

	HepG2 WT		HepG2 KO		Huh7 WT		Huh7 KO	
	BSA	PA	BSA	PA	BSA	PA	BSA	PA
ACTIN	1	1	1	1	1	1	1	1
PGAM5	1.13927	1.60946	0	0	0.770128	0.945156	0	0

Supplementary Figure S2. PGAM5 and Actin Expression in HepG2 and Huh7 cells. **A.** Protein expression of Actin (42 kDa) is displayed on the immunoblot (left). The bright field image of the top PVDF membrane is displayed on the right. **B.** Protein expression of PGAM5 (28 kDa) is displayed on the immunoblot (left). **C.** The bright field image of the assembled PVDF membrane is displayed on the right. BSA: bovine serum albumin, PA: palmitate. WT: wild type, KO: PGAM5 KO

A

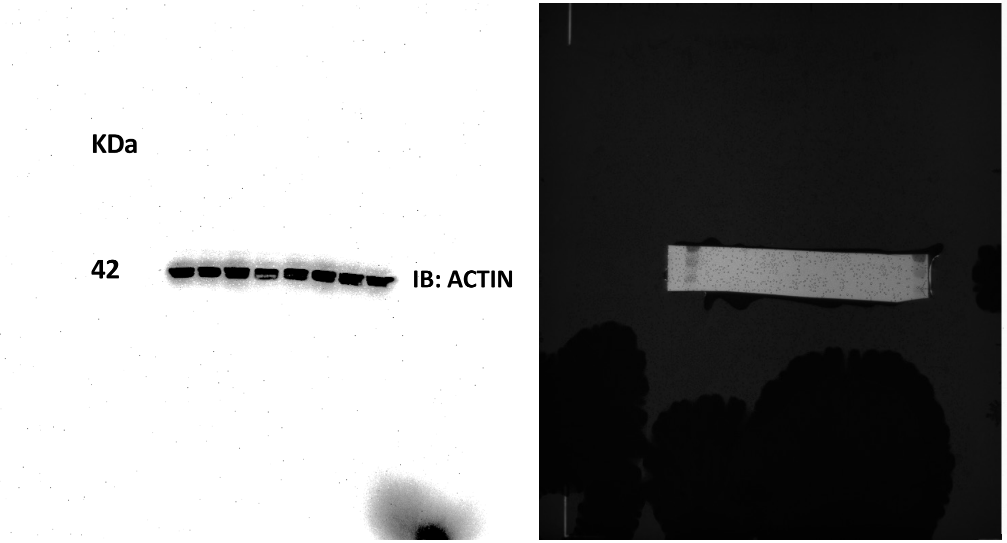

B

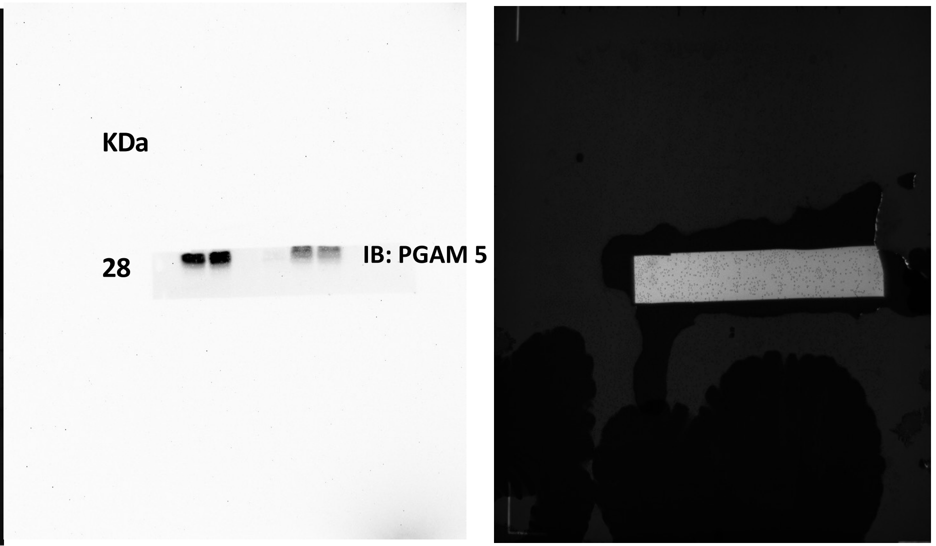

C

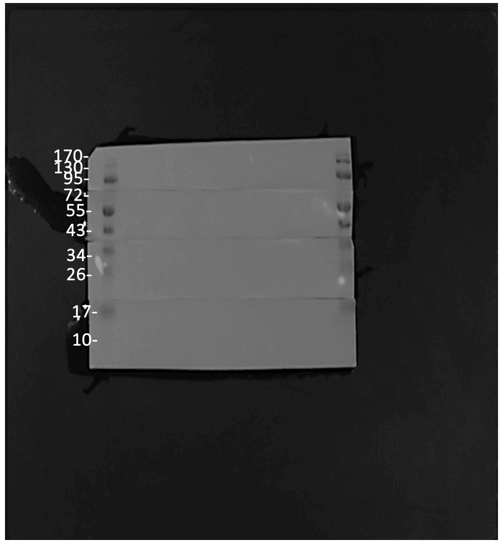

Lane 1 – Marker

Lane 2 – HepG2 WT – BSA

Lane 3 - HepG2 WT – PA

Lane 4 - HepG2 – PGAM5 KO - BSA

Lane 5 - HepG2 – PGAM5 KO - PA

Lane 6 – Huh7 WT – BSA

Lane 7 - Huh7 WT - PA

Lane 8 – Huh7 – PGAM5 KO – BSA

Lane 9 – Huh7 – PGAM5 KO – PA

Lane 10 - Marker

Densitometry Readings

	HepG2 WT		HepG2 KO		Huh7 WT		Huh7 KO	
	BSA	PA	BSA	PA	BSA	PA	BSA	PA
ACTIN	7135.933	5849.205	8035.539	6980.811	7878.711	6835.669	5507.861	4935.054
PGAM5	9162.861	9712.376	0	0	4411.79	4445.104	0	0

Relative Intensities

	HepG2 WT		HepG2 KO		Huh7 WT		Huh7 KO	
	BSA	PA	BSA	PA	BSA	PA	BSA	PA
ACTIN	1	1	1	1	1	1	1	1
PGAM5	1.284	1.660	0	0	0.559	0.650	0	0

Supplementary Figure S3. PGAM5 and Actin Expression in HepG2 and Huh7 cells. **A.** Protein expression of Actin (42 kDa) is displayed on the immunoblot (left). The bright field image of the top PVDF membrane is displayed on the right. **B.** Protein expression of Actin (42 kDa) is displayed on the immunoblot (left). **C.** The bright field image of the assembled PVDF membrane is displayed on the right. BSA: bovine serum albumin, PA: palmitate. WT: wild type, KO: PGAM5 KO

A

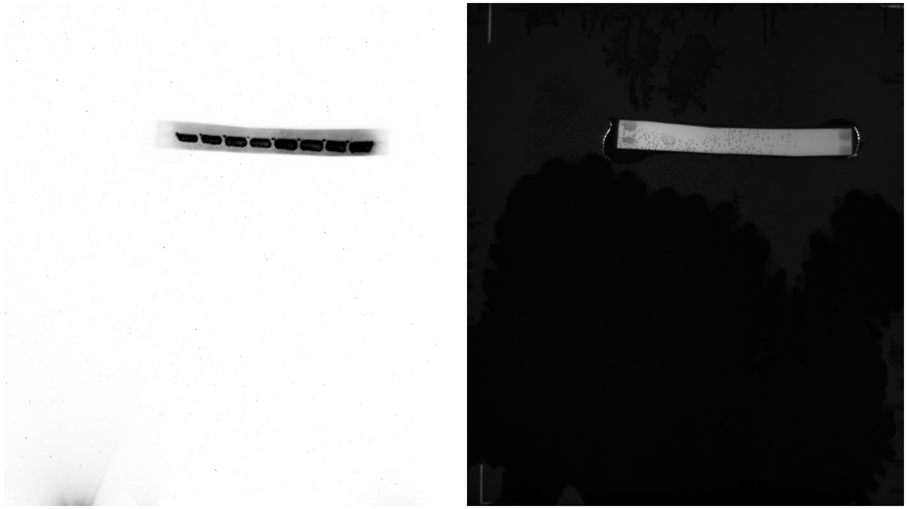

B

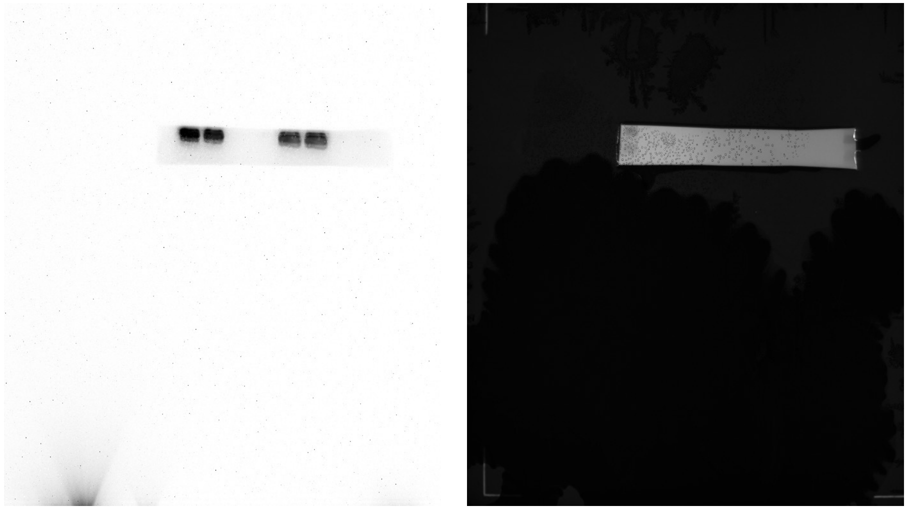

C

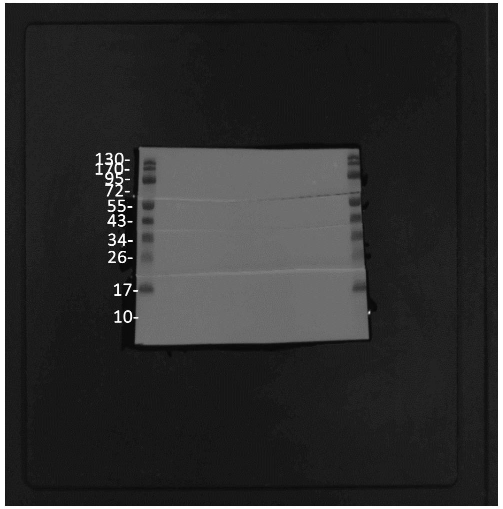

Lane 1 – Marker
Lane 2 – HepG2 WT – BSA
Lane 3 - HepG2 WT – PA
Lane 4 - HepG2 – PGAM5 KO - BSA
Lane 5 - HepG2 – PGAM5 KO - PA
Lane 6 – Huh7 WT – BSA
Lane 7 - Huh7 WT - PA
Lane 8 – Huh7 – PGAM5 KO – BSA
Lane 9 – Huh7 – PGAM5 KO – PA
Lane 10 - Marker

Densitometry Readings

	HepG2 WT		HepG2 KO		Huh7 WT		Huh7 KO	
	BSA	PA	BSA	PA	BSA	PA	BSA	PA
**ACTIN**	5190.397	4826.79	5550.74	3440.69	4470.933	3454.062	3157.598	3813.811
**PGAM5**	8169.841	7926.912	0	0	4421.962	4313.74	0	0

Relative Intensities

	HepG2 WT		HepG2 KO		Huh7 WT		Huh7 KO	
	BSA	PA	BSA	PA	BSA	PA	BSA	PA
**ACTIN**	1	1	1	1	1	1	1	1
**PGAM5**	1.574	1.642	0	0	0.98907	1.24889	0	0

Supplementary Figure S4. CD36, PGAM5, Actin, and FABP1, Expression in HepG2 and Huh7 cells. Protein expression of CD36 (88kDa), Actin (42 kDa), PGAM5 (28 kDa), and FABP1 (17 kDa) are displayed on the assembled immunoblot (left). The bright field image of the PVDF membrane is displayed in the middle panel. The densitometric readings and relative intensities are displayed in the right panel.

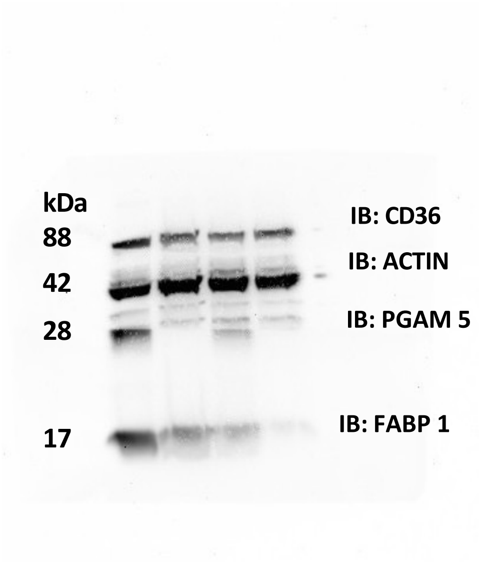

Lane 1 – Marker
Lane 2 – HepG2 WT
Lane 3 – HepG2 – PGAM5 KO
Lane 4 – Huh7 WT
Lane 5 – Huh7 – PGAM5 KO

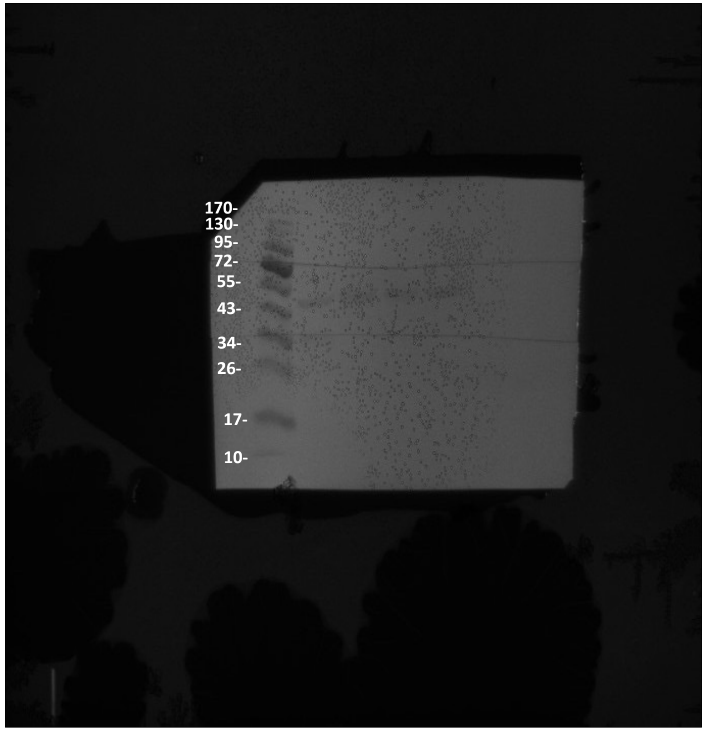

Densitometry Readings

	HEPG2		HUH7	
	WT	PGAM5 KO	WT	PGAM5 KO
**CD36**	12871.9	11785.61	7737.033	8703.711
**PGAM5**	21954.59	0	12243.05	0
**FABP1**	11619.34	3108.489	1968.054	500.678
**ACTIN**	17140.43	16728.37	12736.97	11941.76

Relative Intensities

	HEPG2		HUH7	
	WT	PGAM5 KO	WT	PGAM5 KO
**CD36**	0.750967	0.704528	0.607447	0.728847
**PGAM5**	1.280866	-	0.961222	-
**FABP1**	0.677891	0.185821	0.154515	0.041927
**ACTIN**	1	1	1	1

Supplementary Figure S5. CD36, SLC27A2, PGAM5, Actin, and FABP1 Expression in HepG2 and Huh7 cells. **A.** Protein expression of CD36 (88kDa), SLC27A2 (70 KDa), Actin (42 kDa) and PGAM5 (28 kDa) are displayed on the immunoblot. **B.** Protein expression of FABP1 (14 kDa) is displayed on the middle panel. **C.** The bright field image of the assembled PVDF membrane is displayed on the right panel.

A

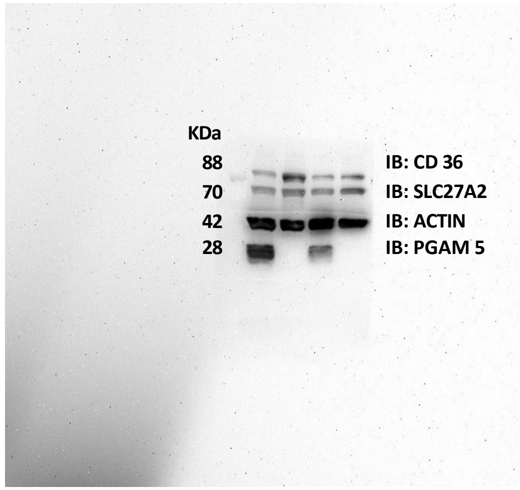

Lane 1 – Marker
Lane 2 – HepG2 WT
Lane 3 – HepG2 - PGAM5 KO
Lane 4 – Huh7 WT
Lane 5 – Huh7 – PGAM5 KO

B

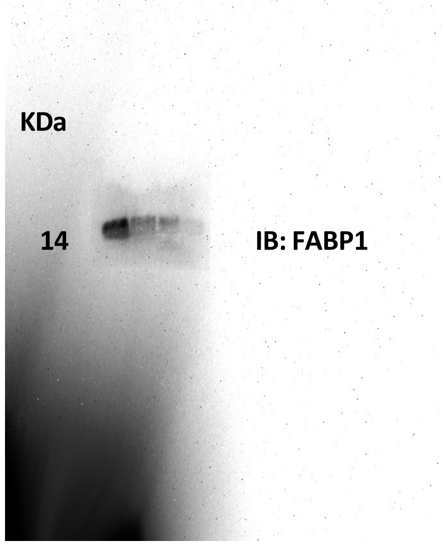

C

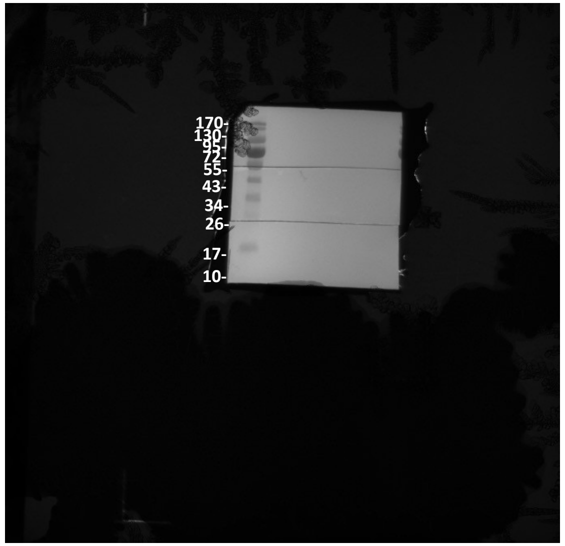

Densitometry Readings

	HEPG2		HUH7	
	**WT**	**PGAM5 KO**	**WT**	**PGAM5 KO**
**CD36**	9430.489	9251.054	8330.225	10158.76
**SLC27A2**	5376.167	4594.267	0	0
**PGAM5**	25468.27	0	19374.49	0
**FABP1**	9530.622	2530.602	2478.075	885.77
**ACTIN**	14993.19	13303.28	11530.54	10125.39

Relative Intensities

	HEPG2		HUH7	
	**WT**	**PGAM5 KO**	**WT**	**PGAM5 KO**
**CD36**	0.628985	0.695397	0.722449	1.003296
**SLC27A2**	0.68633	0.6801	0.638457	0.964528
**FABP1**	0.374216	0.190224	0.214914	0.08748
**ACTIN**	1	1	1	1

Supplementary Figure S6. Actin, SLC27A2, and FABP1 Expression in HepG2 and Huh7 cells. A Protein expression of Actin (42 kDa) is displayed on the left half of the immunoblot (left panel). The bright field images of the PVDF membrane is displayed on the right panel. **B** Protein expression of SLC27A2 (70 kDa) and FABP1 (17 kDa) are displayed on the right half of the immunoblot (left panel). The bright field images of the PVDF membrane is displayed on the right panel. The densitometric readings and relative intensities are displayed in the bottom panel.

A

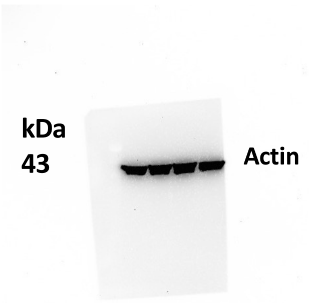

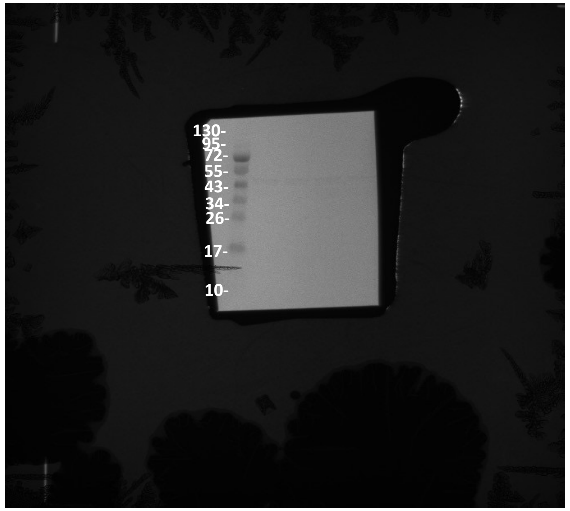

Lane 1 – Marker
Lane 2 – HepG2 WT
Lane 3 – HepG2 – PGAM5 KO
Lane 4 – Huh7 WT
Lane 5 – Huh7 – PGAM5 KO

B

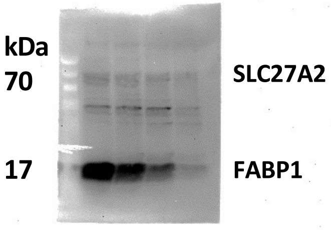

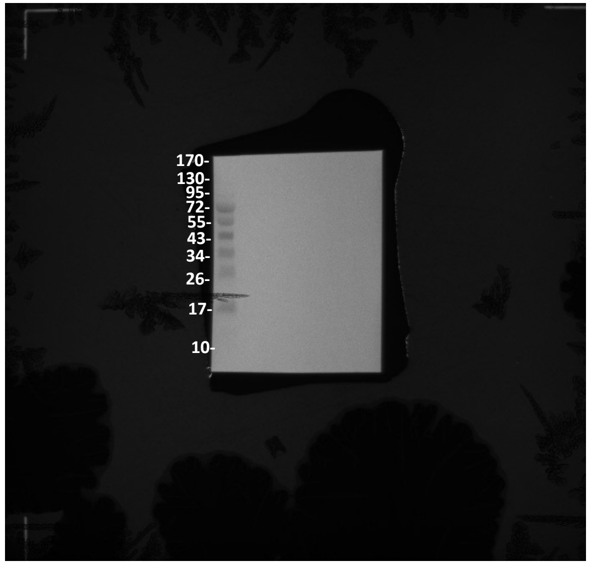

Lane 1 – Marker
Lane 2 – HepG2 WT
Lane 3 – HepG2 – PGAM5 KO
Lane 4 – Huh7 WT
Lane 5 – Huh7 – PGAM5 KO

Densitometry Readings

	HEPG2		HUH7	
	WT	PGAM5 KO	WT	PGAM5 KO
SLC27A2	1812.77	1686.991	1106.92	1190.092
FABP1	3263.296	1521.619	1254.033	503.234
ACTIN	1702.376	1824.648	2468.376	2522.891

Relative Intensities

	HEPG2		HUH7	
	WT	PGAM5 KO	WT	PGAM5 KO
SLC27A2	1.064847	0.924557	0.448441	0.471718
FABP1	1.916907	0.833925	0.50804	0.199467
ACTIN	1	1	1	1

Supplementary Figure S7. SLC27A5 and Actin Expression in HepG2 and Huh7 cells. Protein expression of SLC27A5 (75.4 kDa) and Actin (42 kDa) are displayed on the assembled immunoblot (left panels). The bright field images of the PVDF membrane is displayed on the right panels.

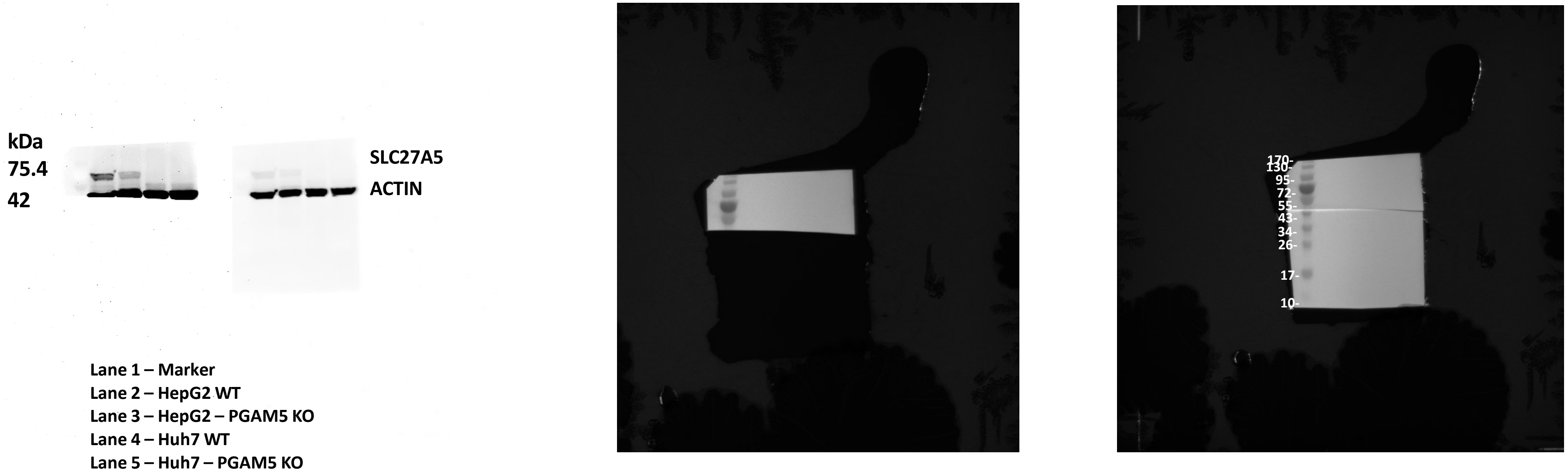

Densitometry Readings

	HEPG2		HUH7	
	WT	PGAM5 KO	WT	PGAM5 KO
SLC27A5	6540.447	3342.548	0	0
ACTIN	3932.477	4289.426	6404.648	6373.062

Relative Intensities

	HEPG2		HUH7	
	WT	PGAM5 KO	WT	PGAM5 KO
SLC27A5	1.663188	0.779253	0	0
ACTIN	1	1	1	1

Supplementary Figure S8. SLC27A5, and Actin, Expression in HepG2 and Huh7 cells. **A.** Protein expression of SLC27A5 (75.2 kDa) is displayed on the immunoblot (left). The bright field image of the top PVDF membrane is displayed on the right. **B.** Protein expression of ACTIN (42 kDa) is displayed on the assembled immunoblot (left). **C.** The bright field image of the assembled PVDF membrane is displayed on the right.

A

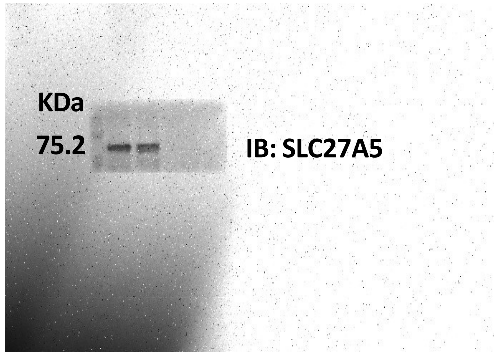

Lane 1 – Marker
Lane 2 – HepG2 WT
Lane 3 – HepG2 - PGAM5 KO
Lane 4 – Huh7 WT
Lane 5 – Huh7 – PGAM5 KO

B

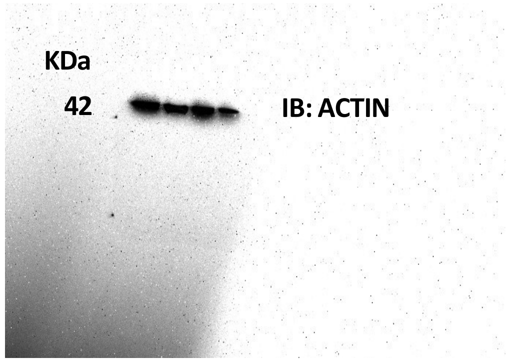

C

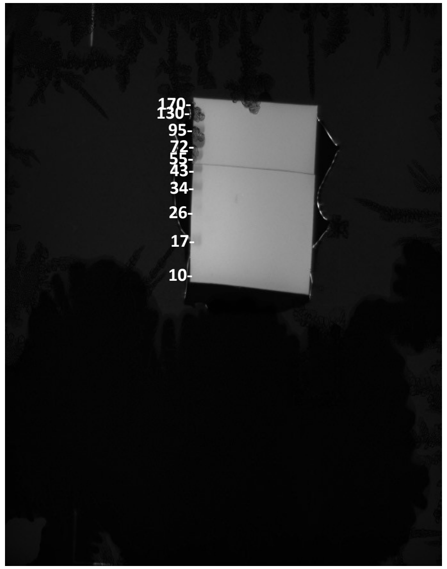

Densitometry Readings

	HEPG2		HUH7	
	WT	PGAM5 KO	WT	PGAM5 KO
SLC27A5	2482.912	2084.255	0	0
ACTIN	4128.355	3525.355	3246.062	1549.74

Relative Intensities

	HEPG2		HUH7	
	WT	PGAM5 KO	WT	PGAM5 KO
SLC27A5	0.601429	0.591218	0	0
ACTIN	1	1	1	1

Supplementary Figure S9. SLC27A5 and Actin Expression in HepG2 and Huh7 cells. **A.** Protein expression of SLC27A5 (75.2 kDa) is displayed on the immunoblot (left). The bright field image of the top PVDF membrane is displayed on the right panel. **B.** Protein expression of ACTIN (42 kDa) is displayed on the immunoblot (left). The bright field image of the bottom PVDF membrane is displayed on the right panel. **C.** The bright field image of the assembled PVDF membrane is displayed. The densitometric readings and relative intensities are displayed in the bottom panel.

A

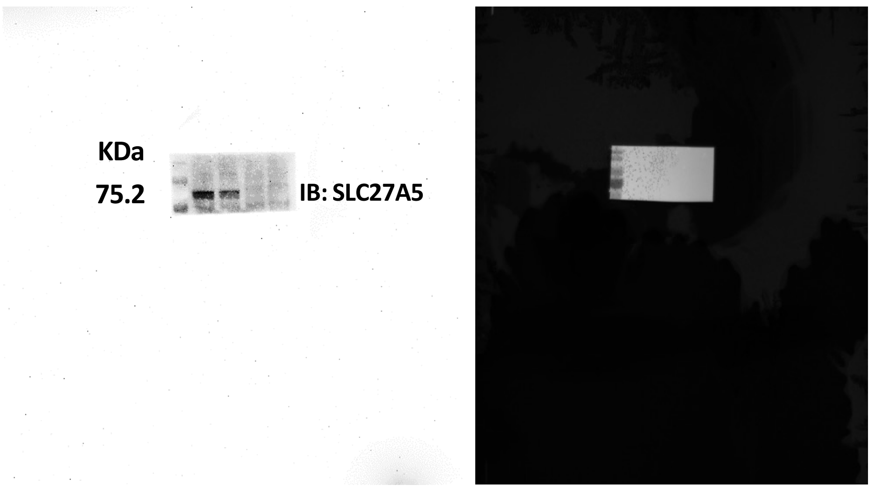

B

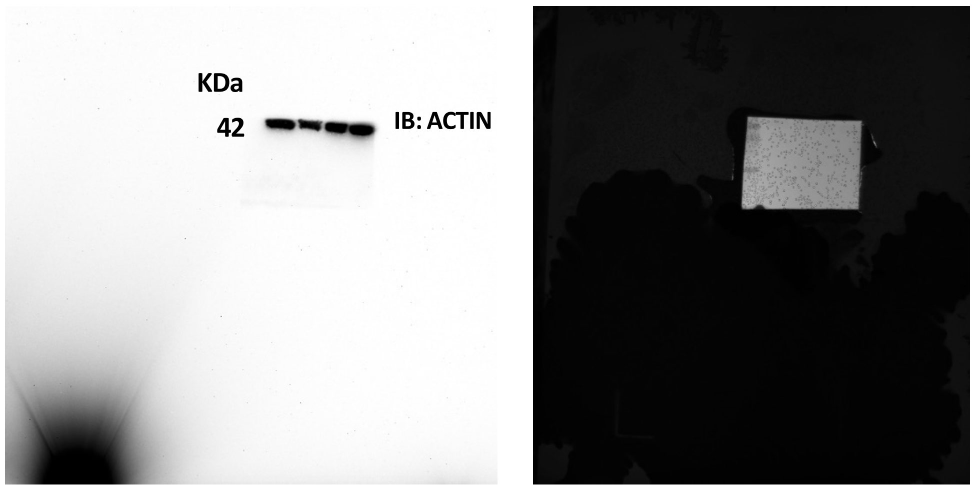

C

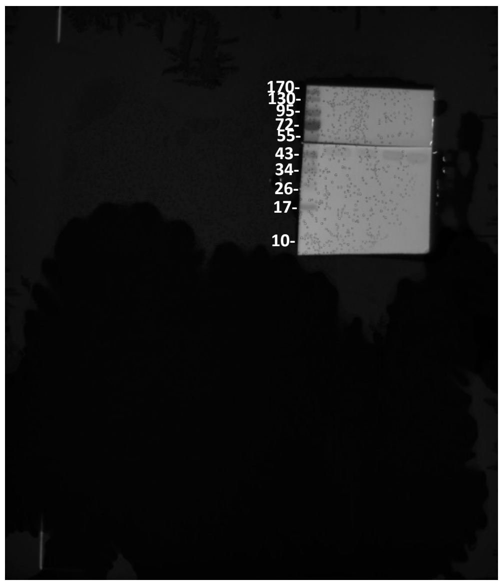

Lane 1 – Marker
Lane 2 – HepG2 WT
Lane 3 – HepG2 – PGAM5 KO
Lane 4 – Huh7 WT
Lane 5 – Huh7 – PGAM5 KO

Densitometry Readings

	HEPG2		HUH7	
	**WT**	**PGAM5 KO**	**WT**	**PGAM5 KO**
**SLC27A5**	3882.648	2247.234	0	0
**ACTIN**	6110.255	4007.406	2726.276	2956.326

Relative Intensities

	HEPG2		HUH7	
	**WT**	**PGAM5 KO**	**WT**	**PGAM5 KO**
**SLC27A5**	0.635431	0.56077	0	0
**ACTIN**	1	1	1	1

Supplementary Figure S10. CD36 and Actin Expression in HepG2 and Huh7 cells. Protein expression of CD36 (88 kDa) and Actin (42 kDa) are displayed on the assembled immunoblot (left panel). The bright field images of the PVDF membrane is displayed on the middle panel. The densitometric readings and relative intensities are displayed in the right panel.

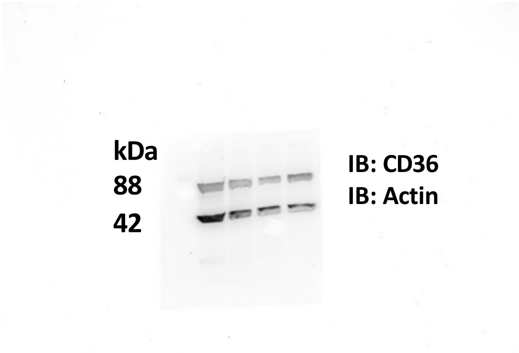

Lane 1 – Marker
Lane 2 – HepG2 WT
Lane 3 – HepG2 – PGAM5 KO
Lane 4 – Huh7 WT
Lane 5 – Huh7 – PGAM5 KO

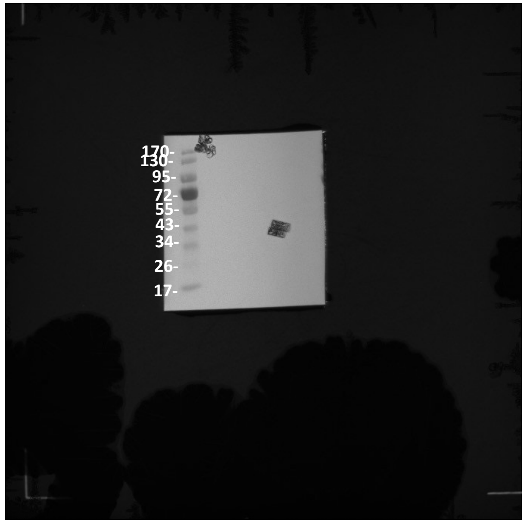

Densitometry Readings

	HEPG2		HUH7	
	WT	PGAM5 KO	WT	PGAM5 KO
**CD36**	4077.305	2171.255	1861.042	3114.87
**ACTIN**	7553.598	4029.104	3437.134	3915.912

Relative Intensities

	HEPG2		HUH7	
	WT	PGAM5 KO	WT	PGAM5 KO
**CD36**	0.539783	0.538893	0.541452	0.795439
**ACTIN**	1	1	1	1

Supplemental Table S1. Quantitative PCR primers

Gene Symbol	qPCR Primer F, 5'-3'	qPCR Primer R, 5'-3'
PGAM5	*TCTTCATCTGTCACGCCAAC*	*AGCACAAAATCCCCAGCG*
CD36	*CAGGTCAACCTATTGGTCAAGC*	*GCCTTCTCATCACCAATGGTCC*
FABP1	*GGAGGAATGTGAGCTGGAGACA*	*TATGTCGCCGTTGAGTTCGGTC*
SLC27A2	*GTGGAGAAAGATGAACCTGTCCG*	*CTGAGCCTTTGCTCCAGCATAG*
SLC27A5	*GGAAGTCTACGGCTCCACAGAA*	*GTCGAACTGCACCAGCTCAAAG*
ß-Actin	*CATCTCTTGCTCGAAGTCCA*	*ATCATGTTTGAGACCTTCAACA*
18s	*GGATTGACAGATTGATAGC*	*TATCGGAATTAACCA GACAA*

Supplemental Table S2. Individual qPCR data points corresponding to Figure 3

HepG2 cells

Gene Symbol	Experiment _Replicate	Wildtype -ddCT	*PGAM5* KO -ddCT
CD36	1_1	-0.32	-0.77
	1_2	0.32	-0.81
	2_2	0.026	-3.49
	2_2	-0.026	-3.05
	3_1	0.64	-0.43
	3_2	-0.64	-0.69

Gene Symbol	Experiment _Replicate	Wildtype -ddCT	*PGAM5* KO -ddCT
SLC27A2	1_1	0.004	-0.07
	1_2	0.004	0.10
	2_2	-0.11	-0.51
	2_2	0.11	0.01
	3_1	0.54	-0.63
	3_2	-0.54	-0.64

Gene Symbol	Experiment _Replicate	Wildtype -ddCT	*PGAM5* KO -ddCT
SLC27A5	1_1	0.19	-0.08
	1_2	-0.19	-0.12
	2_2	0.08	-1.15
	2_2	-0.08	-1.02
	3_1	0.63	-0.71
	3_2	-0.63	-1.08

Gene Symbol	Experiment _Replicate	Wildtype -ddCT	*PGAM5* KO -ddCT
FABP1	1_1	0.27	-1.15
	1_2	-0.27	-1.62
	2_2	-0.14	-0.80
	2_2	0.14	-0.70
	3_1	0.78	-1.41
	3_2	-0.78	-1.74

Supplemental Table S3. Individual qPCR data points corresponding to Figure 3

Huh7 cells

Gene Symbol	Experiment _Replicate	Wildtype -ddCT	*PGAM5* KO -ddCT
CD36	1_1	-0.05	0.32
	1_2	0.05	0.74
	2_2	-0.18	-0.43
	2_2	0.18	-2.08
	3_1	0.04	0.45
	3_2	-0.04	-0.27

Gene Symbol	Experiment _Replicate	Wildtype -ddCT	*PGAM5* KO -ddCT
SLC27A2	1_1	-0.20	-0.30
	1_2	0.20	-0.16
	2_2	0.24	0.45
	2_2	-0.24	0.37
	3_1	0.13	0.84
	3_2	-0.13	0.46

Gene Symbol	Experiment _Replicate	Wildtype -ddCT	*PGAM5* KO -ddCT
SLC27A5	1_1	0.05	-0.81
	1_2	-0.05	-0.68
	2_2	0.01	-0.21
	2_2	-0.01	-0.11
	3_1	0.01	0.68
	3_2	-0.01	0.64

Gene Symbol	Experiment _Replicate	Wildtype -ddCT	*PGAM5* KO -ddCT
FABP1	1_1	-0.21	-0.11
	1_2	0.21	0.34
	2_2	-0.02	-4.02
	2_2	0.02	-3.66
	3_1	-0.08	-2.59
	3_2	0.08	-2.32
